# Predictive machine learning model for 30-day hospital readmissions in a tertiary healthcare setting

**DOI:** 10.1093/bioadv/vbaf121

**Published:** 2025-05-24

**Authors:** Diego Halac, Cecilia Cocucci, Sebastian Camerlingo

**Affiliations:** Departamento de Medicina Interna, Sanatorio Anchorena de San Martin, Perdriel 4189, CP: 1650. Gral. San Martin, Provincia de Buenos Aires, Argentina; Instituto de Efectividad Clinica y Sanitaria (IECS), Dr. Emilio Ravignani 2024, CP:1414 CABA, Argentina; Catedra de Bioestadistica, Hospital universitario Austral, Avenida Juan Domingo Perón 1500, Pilar CP: B1629AHJ, Provincia de Buenos Aires, Argentina; Instituto de Efectividad Clinica y Sanitaria (IECS), Dr. Emilio Ravignani 2024, CP:1414 CABA, Argentina; Catedra de Bioestadistica, Hospital universitario Austral, Avenida Juan Domingo Perón 1500, Pilar CP: B1629AHJ, Provincia de Buenos Aires, Argentina; Instituto de Efectividad Clinica y Sanitaria (IECS), Dr. Emilio Ravignani 2024, CP:1414 CABA, Argentina; Catedra de Bioestadistica, Hospital universitario Austral, Avenida Juan Domingo Perón 1500, Pilar CP: B1629AHJ, Provincia de Buenos Aires, Argentina

## Abstract

**Motivation:**

Hospital readmissions represent a major challenge for healthcare systems due to their impact on patient outcomes and associated costs. As many readmissions are considered preventable, predictive modeling offers a valuable tool for early identification and intervention. This study aimed to develop and validate a predictive model for 30-day readmissions in a 200-bed community hospital in Argentina. A retrospective analysis was conducted on 3388 adult admissions. The primary endpoint was readmission within 30 days of discharge. Predictor variables included demographic and clinical factors such as age, length of stay, hypertension, diabetes, heart failure, coronary artery disease, stroke, cancer, dementia, chronic kidney disease, chronic obstructive pulmonary disease, and bedridden status. Three models—Logistic Regression (LR), Random Forest (RF), and LightGBM (LGBM)—were developed, with hyperparameter tuning via Bayesian optimization. Model performance was assessed using calibration, discrimination (C-statistics), and decision curve analysis. Internal validation was performed using 250 bootstrap resamples.

**Results:**

The readmission rate was 11% (*n* = 394). RF outperformed LR and LGBM in discrimination and clinical utility within predictive probability thresholds of 0.05–0.25. Optimism-corrected C-statistics were 0.60 (LR, LGBM) and 0.64 (RF); calibration slopes were 0.75 (LR), 1.13 (RF), and 1.76 (LGBM). Machine learning models, particularly RF, can improve readmission risk prediction and inform targeted healthcare interventions.

**Availability and implementation:**

The dataset and code used to develop and validate the predictive models are available from the corresponding author upon reasonable request. The implementation was conducted in R using the mlr3verse, pminternal, rms, dcurves, data.table, tidyverse, ranger and lightgbm packages, with Bayesian hyperparameter optimization via mlr3mbo.

## 1 Introduction

Hospital readmission is a significant concern due to its impact on patient outcomes and healthcare costs. Although variable across institutions and regions, in the US Medicare patients have a 30-day readmission rate of 20%. Of this, 12% is considered preventable, representing a potential savings of $1 billion (Rosso 2021). Differentiating between avoidable and unavoidable readmissions remains challenging in clinical settings. However, recent United States initiatives to incentivize hospitals to reduce readmission rates show promise in improving healthcare quality (Rosso 2021). Similarly, the United Kingdom has reported an increase in readmissions over the last decade, marking the need for predictive tools to identify those at-risk patients (McIlvennan and Allen 2018, The Health Foundation 2021). In Argentina, scarce and low-quality data about hospital readmissions is available. However, it aligns with international reports, which indicate rates between 11% and 18%, with 25% attributed to avoidable causes, and a 10% increase in rates within the public health system. Therefore, predicting hospital readmissions becomes a cornerstone for health management in Argentina, particularly in the public healthcare system, where resources are limited, and avoidable readmissions are more frequent (Muñoz-Cruzado *et al.* 2010, Centeno 2015, Giunta *et al.* 2020, Chavesta Carrillo and Lucero 2023).

Hospital readmissions pose a dual threat to both patient health and healthcare system performance, and therefore, applying predictive tools and targeted interventions may address these issues effectively. Although key questions regarding feasibility and cost-effectiveness of implementing predictive models and targeted follow-up programs for high-risk patients need to be evaluated, identifying these patients upon discharge will allow more efficient resource allocation to prevent readmissions.

Developing predictive models based on clinical and demographic factors is essential to early detect high-risk patients and implement targeted interventions, improve patient outcomes and optimize resource utilization in healthcare systems. Creating a user-friendly tool for physicians to access readmission risk stratification would significantly enhance value-based medical management.

The aim of this study was to develop a predictive model to assess the risk of readmission within 30 days in a 200-bed community hospital in Argentina.

## 2 Methods

### 2.1 Study population and data collection

Adult individuals aged equal or more than16 years who were discharged alive following a minimum 24-hour admission to a general inpatient ward within a 200-bed community hospital between January 1 and December 31 of 2019 were consecutively enrolled for the study purpose. Administrative, demographic, and clinical data were collected through retrospective examination of Electronic Health Records (EHRs) (Fig. S1, available as supplementary data at *Bioinformatics Advances* online).

### 2.2 Ethical considerations and reporting guidelines

This study was approved by the ethics committee of the HIGA Eva Peron (San Martin, Buenos Aires) approval and performed in accordance with the Declaration of Helsinki and its later amendments (ID: IF-2022–42835601-GDEBA-CECMSALGP). Informed consent was deemed not necessary. Reporting has been performed according to the transparent guidance for multivariable prediction models on individual prognosis or diagnosis that use regression or machine learning methods (TRIPOD+AI) (Collins *et al.* 2024a).

### 2.3 Outcome and candidate predictors

We defined the primary outcome as the presence or absence of readmission within 30 days after discharge from the initial hospitalization. Candidate predictors that were included due to their proven utility in previous studies were: Length of stay measured in days (LOS), age measured in years (AGE), hypertension (HTN), diabetes (DM), heart failure (HF), coronary artery disease (CAD), history of stroke (STROKE), active oncologic disease (CA), DEMENTIA, prostration (BEDRIDDEN), chronic kidney disease (CKD), chronic pulmonary obstructive disease (COPD) (Charlson *et al.* 1987, Donzé *et al.* 2016, Robinson and Hudali 2017, Wadhera *et al.* 2019, Liu *et al.* 2020).

### 2.4 Statistical analysis

#### 2.4.1 Class imbalance handling

Our sample shows class imbalance, with an 11% outcome prevalence. We did not apply class imbalance correction techniques due to the risk of introducing bias and compromising external validity. As discussed by van den Goorbergh *et al.* (2022) and Tarawneh *et al.* (2022), such methods can distort predictor-outcome associations, reduce generalizability, and lead to overfitting. Therefore, we evaluated model performance using the original data distribution to ensure clinical applicability.

#### 2.4.2 Sample size estimation

Sample size calculation was guided by a targeted objective Area Under the Receiver Operating Characteristic Curve (C-statistic) of 0.7, with an expected outcome prevalence of 11%, following the methodology proposed by Reily *et al.* in 2020 (Riley *et al.* 2020), which led to a minimum sample size of 2303 observations and 19 events per predictor (EPP) required.

All statistical analysis was performed with R software version 4.3.2 and RStudio version 2023.12.1.402 (R Core Team 2023, Posit Team 2024).

#### 2.4.3 Model development and validation

We fitted a logistic regression (LR) (Hosmer and Lemeshow 2000), a Random Forest (RF) (Breiman 2001), and a LightGBM (LGBM) (Goulin *et al.* 2017) model including all candidate predictors.

LR is a statistical method used for binary classification, modeling the probability that an observation belongs to a given class. It estimates the relationship between predictor variables and the log-odds of the outcome using a linear function and applies the sigmoid function to produce probabilities (Hosmer and Lemeshow 2000). RF is an ensemble learning method that builds multiple decision trees and combines their predictions to improve accuracy and reduce overfitting. Each tree is trained on a random subset of data using bootstrapping, and feature selection is randomized to increase model diversity. The final prediction is obtained through majority voting, making it a robust and interpretable algorithm (Breiman 2001). LGBM is a gradient boosting framework optimized for speed and efficiency. It constructs decision trees in a leaf-wise manner, allowing it to handle large datasets with high-dimensional features more effectively than traditional boosting methods. LightGBM uses histogram-based learning, which reduces memory usage and speeds up training. It is well-suited for handling categorical variables and capturing complex, non-linear relationships in the data (Goulin *et al.* 2017).

For LR, continuous predictors were modeled using restricted cubic splines with default settings, including four knots positioned at recommended percentiles with the “*rms*” R software package (Harrell 2023). The RF and the LGBM models underwent hyperparameter tuning through Bayesian optimization (C-Statistic maximization criteria) (Shahriari *et al.* 2016) for hyperparameters: *mtry*, *num_trees*, and max_depth for RF and *learning_rate*, *feature_fraction*, *min_data_in_leaf*, and *num_leaves* for LGBM. All models were trained on the full original dataset and validated using bootstrapping (*n* = 250 samples).

#### 2.4.4 Models’ performance and calibration assessment

After predictive models’ fitting, we conducted a comparative analysis between them. Model performance and calibration were evaluated with C-Statistic, F2-Score, calibration slopes and flexible curves (Steyerberg 2019, Van Calster *et al.* 2019), Eavg [Integrated Calibration Index (ICI)], and E50th percentile (E50) metrics.

Clinical utility was quantified as net benefit (Vickers *et al.* 2016). Model’s discrimination was reported with C-statistics, which varies from 0.5 to 1, where 0.5–0.7 indicates good, 0.7–0.8 strong, and 1 perfect discrimination. It estimates the likelihood that a randomly selected hospitalization with a high predicted probability of readmission will effectively result in a readmission. For binary outcomes, C-statistics works similarly to the area under the receiver operating characteristics curve (AUC-ROC). The DeLong test and the bootstrap method were used to compare differences between AUC-ROC values. Additionally, we used the F2 score to compare model performance as a secondary metric by applying a cutoff. The F2 score represents the harmonic mean of positive predictive value (ppv) and sensitivity, emphasizing sensitivity more than ppv by weighting sensitivity twice as heavily. Calibration slope was used to evaluate the spread of the estimated risks. It has a target value of 1 and a slope <1 suggests that estimated risks are too extreme, i.e. too high for hospitalizations who represent high risk and too low for hospitalizations who represent low risk. A slope >1 suggests the opposite, i.e. that risk estimates are too moderate. Ideally, the slope should be close to 1 (Vickers *et al.* 2016). Flexible calibration curves show the relation between the estimated risk (on the *x*-axis) and the observed proportion of events (*y*-axis), i.e. using loess or spline functions. A curve close to the diagonal indicates that predicted risks correspond well to observed proportions (Van Calster *et al.* 2019, De Cock and Campo 2023). Eavg was used to estimate the weighted average absolute difference between predicted probabilities and the occurrence of the outcome, where differences are weighted by the empirical density function of predicted probabilities. An advantage of the proposed metric is that it provides a numeric summary of differences across the entire range of predicted probabilities in a way that accounts for the distribution of predicted probabilities. While mean is a commonly used summary statistic, other metrics can be used to summarize the absolute difference between observed and predicted probabilities. We reported E50 to denote the median absolute difference between observed and predicted probabilities. It is a high-quality metric to compare relative calibration on multiple predictive methods and a close to zero value indicates better calibration. In addition to global calibration and to highlight differences across various risk levels, a stratified calibration analysis by predicted risk level was conducted. All the calibration and prediction stability assessment were performed with the “*pminternal*” R software package (Rhodes 2023).

Finally, Decision Curve Analysis (DCA) was employed to assess clinical utility, weighting the trade-off between correctly identified true positives and false positives based on threshold probability. Curves were smoothed with locally weighted smoothing (LOESS) (Vickers et al. 2016). DCA provided insights into risk-benefit at various probability thresholds, comparing different treatment strategies and model performances. For the best model, we estimated sensitivity, specificity, positive predictive value, and negative predictive value (Altman and Bland 1994) using a probability cutoff that aimed to minimize type II over type I error, thereby reducing misclassifications of high-risk readmissions as non-high-risk while allowing for some false positive misclassifications.

An external validation using new data not available at the time of model development is planned in the future.

## 3 Results

A total of 3388 admissions were included in the analysis with 394 readmission events (11% of total admissions). HTN, HF, Cancer, and CKD were the most frequent hospitalization causes. Table 1 shows these demographic and predictors data distribution.

**Table 1.** Demographics.

	Overall	No readmission	Readmission
*N*	3388	2994	394
Length of stay (LOS) (mean(SD))	6.75 (8.67)	6.58 (8.60)	8.05 (9.12)
AGE (mean(SD))	58.04 (19.10)	57.63 (19.08)	61.11 (18.97)
Hypertension (HTN) (*n*, %)	115 (3.4)	100 (3.3)	15 (3.8)
Diabetes mellitus (DM) (*n*, %)	88 (2.6)	79 (2.6)	9 (2.3)
Heart failure (HF) (*n*, %)	117 (3.5)	97 (3.2)	20 (5.1)
Coronary artery disease (CAD) (*n*,%)	21 (0.6)	15 (0.5)	6 (1.5)
Stroke (*n*, %)	53 (1.6)	51 (1.7)	2 (0.5)
Oncologic disease (CA) (*n*, %)	208 (6.1)	169 (5.6)	39 (9.9)
Dementia (*n*, %)	35 (1)	30 (1)	5 (1.3)
Bedridden (*n*, %)	27 (0.8)	22 (0.7)	5 (1.3)
Chronic kidney disease (CKD) (*n*, %)	376 (11.1)	308 (10.3)	68 (17.3)
Chronic obstructive pulmonary disease (COPD) (*n*, %)	34 (1)	30 (1)	4 (1)

Abbreviations: LOS = length of stay (days), AGE = age (years), HTN = hypertension, DM = diabetes mellitus, HF = heart failure, CAD = coronary artery disease, STROKE = history of stroke, CA = history of oncologic disease, DEMENTIA = cognitive impairment, BEDRIDDEN = prostration, CKD = chronic kidney disease, COPD = chronic obstructive pulmonary disease.

**Table 2.** Model performance.

Model	C-Statistic	Slope	E50	Eavg
Logistic regression	0.60 (0.57–0.63)	0.75 (0.51–1.01)	0.002	0.005
Random forest	0.64 (0.61–0.66)	1.13 (0.83–1.45)	0.005	0.002
LightGBM	0.60 (0.58–0.64)	1.76 (1.28–2.22)	0.014	0.013

Global performance. C-statistic: Refers to optimism adjusted C-statistic (Optimism: Refers to the overestimation of a model’s performance on training data compared to unseen data, typically addressed by validation based on bootstrapping). Eavg: The density of the probabilities weighted average of the absolute difference between the calibration curve of the model and the diagonal line of perfect calibration. E50: The density of the probabilities weighted median of the absolute difference between the calibration curve of the model and the diagonal line of perfect calibration.

C-statistic and its correction for optimism for models’ performance assessment are shown in Tables 2 and 3 along with calibration metrics (calibration slope, E50, and Eavg).

**Figure 1.**
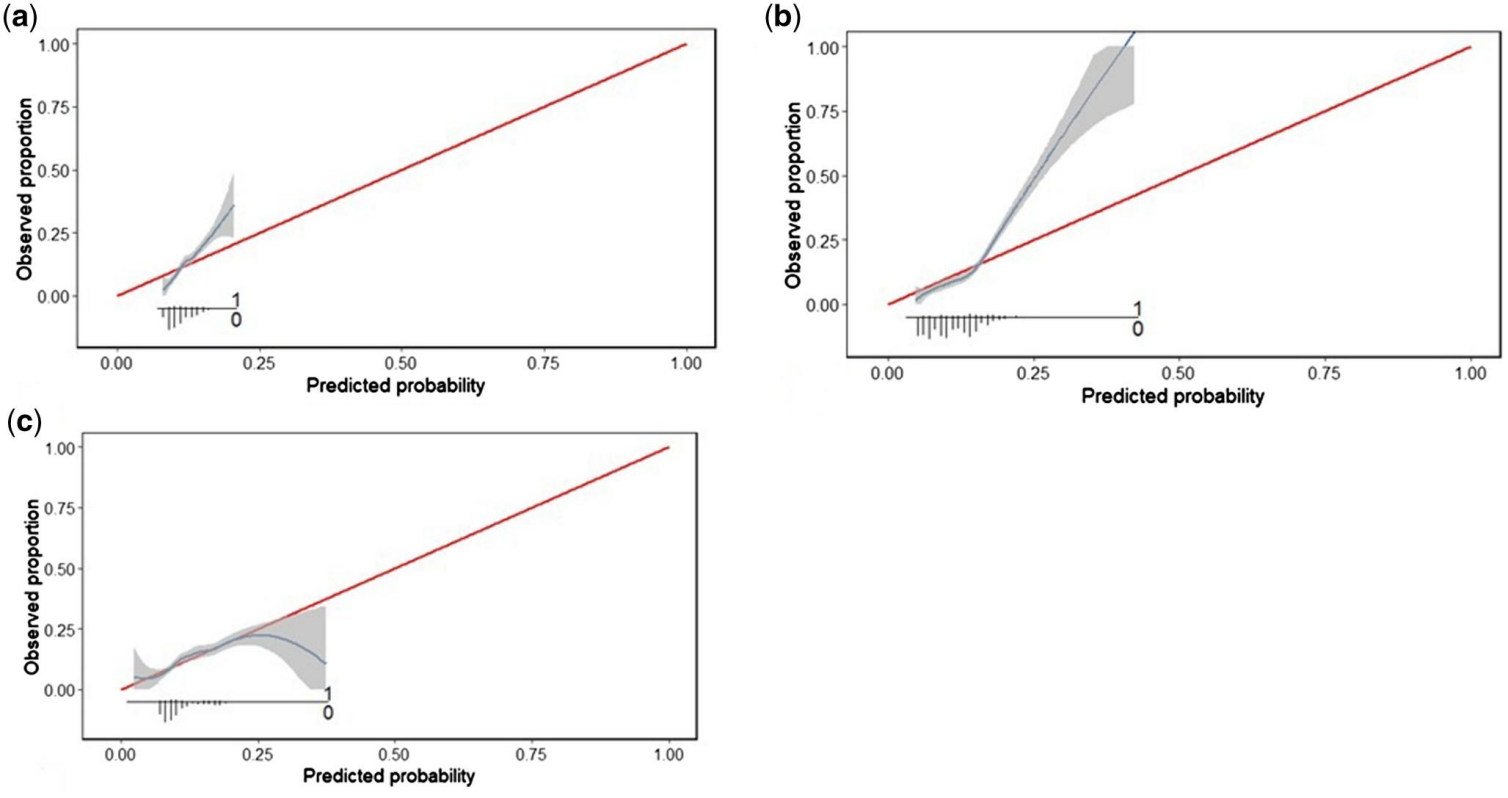
(a) Calibration curve of LightGBM model. (b) Calibration curve of Random Forest model. (c) Calibration curve of Logistic Regression model.

The optimism-corrected calibration slope values for the three models were: 0.75 for LR, 1.13 for RF, and 1.76 for LGBM. Smooth calibration plots are displayed for visual comparison in Fig. 1.

For the selected threshold, the F2 scores were 0.45 for the RF model, 0.39 for LR, and 0.42 for the LGBM model. These results reinforce the selection of the RF model as the best-performing model when evaluated using this secondary metric (Table 3).

**Table 3.** Model performance metrics having selected probability cutoff = 0.10.

Model	F2-Score	Sensitivity	Specificity	PPV	NPV
Logistic regression	0.39	0.66	0.5	0.92	0.15
Random forest	0.45	0.82	0.44	0.95	0.16
LightGBM	0.42	0.86	0.30	0.94	0.14

PPV: Positive predictive value. Indicates the probability that a positive prediction corresponds to an actual positive case. NPV: Negative predictive value. Indicates the probability that a negative prediction corresponds to an actual negative case.

Due to different values observed at various levels of predicted risk, slope values could not be summarized in a unique measure for model calibration, as Fig. 1 shows. Additionally, for risks above 0.20 there were fewer observations and so predictive power was reduced. Thus, metrics that consider the density of observations at each risk level, such as Eavg and E50, were more appropriate for reporting this border effect behavior.

The LR model was prone to overestimating the risk of readmission among those observations with high predicted risk and to calibrate appropriately in those with risk <20%. For both, LGBM, and particularly RF, models underestimated the risk in observations with extreme predicted risk, while at values <20%, calibration was adequate.

The stratified calibration analysis by predicted risk level showed better calibration in intermediate ranges, while significantly poorer calibration was observed in high predicted risk ranges (Table 4). These results reinforced the observations from the global calibration analysis of the three models.

**Table 4.** Model calibration by predicted risk subgroups.

Model	Risk	E50	Eavg
Logistic regression	Low (predicted probability 0–0.10)	0.029	0.030
	Medium (predicted probability 0.10–0.20)	0.020	0.027
	High (predicted probability >0.20)	0.093	0.100
Random forest	Low (predicted probability 0–0.10)	0.035	0.04
	Medium (predicted probability 0.10–0.20)	0.0029	0.007
	High (predicted probability >0.20)	0.360	0.350
LightGBM	Low (predicted probability 0–0.10)	0.018	0.019
	Medium (predicted probability 0.10–0.20)	0.004	0.009
	High (predicted probability >0.20)		

Calibration metrics in low, medium, and high risk strata.

**Figure 2.**
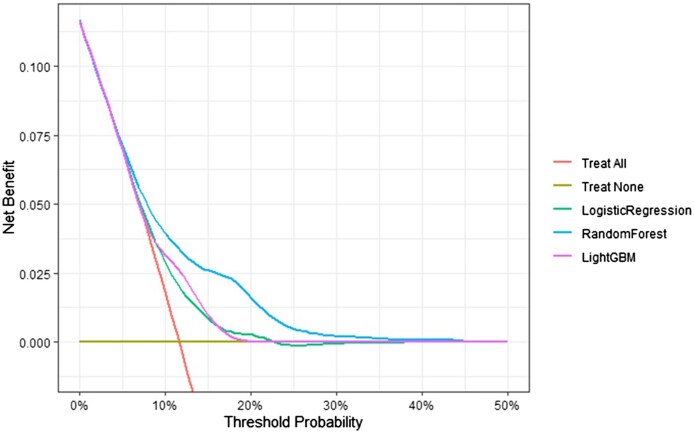
Decision Curve Analysis comparing Random Forest, LightGBM, and Logistic Regression with treat-all and treat-none strategies.

Figure 2 shows the DCA, highlighting the net benefit of utilizing the RF model as a predictive tool compared to the LR and LGBM models, as well as the model utilization for decision-making under “treat-all” and “treat-none” strategies. The probability range where this model provides the greatest contribution is between 0.05 and 0.25, making it a reliable method for identifying potential interventions, as it covers nearly the entire risk range observed in our study population.

Figure S2, available as supplementary data at *Bioinformatics Advances* online shows the Receiver Operating Characteristic (ROC) curves for all three models. Additionally, Fig. S3, available as supplementary data at *Bioinformatics Advances* online presents the sensitivity, specificity, positive predictive value, and negative predictive value across the full range of probability thresholds. Considering the prevalence in our population and the specific problem addressed, our selected cutoff point was 0.10. For the best-performing model (RF), the estimated metrics with this cutoff were sensitivity: 0.82, specificity: 0.44, positive predictive value: 0.16, and negative predictive value: 0.94. The comparison of AUC-ROC was performed using the DeLong test and bootstrap resampling, revealing statistically significant differences in favor of the AUC reported for the RF model.

## 4 Discussion

In this study we developed and validated a predictive model that provides the risk of readmission after a primary hospitalization. Discriminative capacity favored RF model over LGBM and LR models.

In view of the observed class imbalance, when adding the SMOTE algorithm to the model pipeline, we obtained worse performance and calibration compared to not using it (Table S1, available as supplementary data at *Bioinformatics Advances* online).

Our selected predictors align with those reported in the literature. Wadhera *et al.* (2019) examined readmission prediction using variables related to heart failure, myocardial infarction, pneumonia, and demographic factors such as sex, age, and race. Similarly, Wenshuo Liu *et al.* (2020) evaluated predictive model performance using variables from myocardial infarction, pneumonia, and heart failure, incorporating clinical factors like length of stay and hospital expenses for readmission prediction (Liu *et al.* 2020). The HOSPITAL and LACE scores are widely accepted predictors of rehospitalization in the United States. The HOSPITAL score includes clinical variables such as anemia, oncological disease, hyponatremia, surgical procedures during hospitalization, and a hospital stay longer than 5 days. The LACE score combines length of stay with variables from the Charlson Comorbidity Index, which assesses conditions like heart failure, peripheral vascular disease, dementia, stroke, COPD, CKD, diabetes, oncological disease, liver disease, HIV, peptic ulcer disease, and valvular heart disease (Charlson *et al.* 1987, Donzé *et al.* 2016, Robinson and Hudali 2017). In terms of discrimination with C-statistic metric, our best model achieves similar or better performance compared to other models. For instance, Robinson and Hudali (2017) reported a C-statistic of 0.75 for the HOSPITAL score and 0.58 for the LACE score.

One of the most significant strengths of our study is the meticulous validation and calibration process. These techniques enable better comparison between models by utilizing not only the AUC but also the net benefit. However, many studies do not emphasize model calibration, and when it is reported, it often relies on tests like the Pearson goodness-of-fit and the Hosmer and Lemeshow tests, which are criticized for their low power to detect miscalibration (Van Calster *et al.* 2019). Miscalibration in ML models is often linked to a limited number of observations at certain predicted probability levels, leading to inaccurate diagnoses of poor calibration. To address this, our study implemented advanced techniques for validating and calibrating AI models, allowing us to assess optimism in predictions and generate weighted metrics of calibration based on the number of observations at each risk level. Bootstrap-based optimism evaluation mitigates for overfitting as it determines the stability of predictions and corrects performance metrics to obtain more robust measurements. Moreover, Eavg and E50 ICI metrics facilitate comparing each model calibration considering the scant number of observations at specific thresholds.

The clinical translation of these complex metrics focuses on rare and extreme event frequencies, where prediction may be less critical. Eavg(ICI) measures the average absolute difference between predicted probabilities and observed outcomes, evaluating model calibration by considering the distribution density of observations across risk levels. This approach assesses model performance not just overall but at each predicted risk level, offering a detailed and accurate evaluation. Additionally, applying appropriate calibration techniques improves performance evaluation beyond the C-statistic metric. Net benefit is a more informative metric for assessing classification models in real-world settings. Our analysis revealed differences in net benefit and performance when using the RF model compared to the LGBM and LR models, with no significant differences in calibration metrics or increases in optimism. This highlights a model with both adequate performance and high clinical utility (Collins *et al.* 2024b, Riley *et al.* 2024).

When applying prediction models in clinical settings, it is essential to balance the priority between avoiding false negatives and false positives. In our study, we aimed to minimize type II error, focusing on avoiding false negatives by considering F2-score as a secondary metric. This approach accepts a higher rate of false positives to enhance sensitivity, which is crucial for identifying high-risk patients. Consequently, in practice, the probability threshold for categorizing patients as high or low risk of rehospitalization should be set lower. This strategy is vital for optimizing resource allocation and achieving potential cost savings from targeted interventions (Mohr *et al.* 2021).

The performance of our predictive model is relatively low when evaluated from a general machine learning perspective. However, considering the characteristics of the problem analyzed and the training data, which accurately reflect real-world conditions, the model provides predictions that contribute to solving a significant challenge in daily practice. Moreover, the model development and validation pipeline is considerably more robust than in other studies, where the importance of thoroughly assessing model calibration—beyond traditional performance metrics—is often overlooked.

Our study has several limitations. Incorporating initial or baseline diagnostics as predictors, as done in other studies, could have improved our models’ performance. Additionally, while a perfectly calibrated model is ideal, none of ours achieved this. Despite this, a miscalibrated model can still be clinically useful. In our analysis, although the discrimination metrics (C-statistic) align with similar studies, imperfect calibration might misrepresent model performance. Notably, miscalibration occurred in all three models at high predicted risk levels (>0.20), where such elevated risk levels are rare. Thus, accurately interpreting model evaluation results is crucial for leveraging these models effectively in real-life scenarios (Riley *et al.* 2024). Compared to this study, other models may report C-statistic without thoroughly evaluating calibration or addressing overfitting through metrics correction, potentially leading to misleading conclusions about their clinical applicability.

Black box ML models often exhibit superior AUC compared to traditional regression models. However, their clinical utility hinges on proper validation, calibration, and performance evaluation. Our study highlights the importance of using predictive models that not only identify high-risk patients at discharge but also allow healthcare providers to allocate resources efficiently and implement preventive measures to reduce readmissions. This underscores the value of advanced evaluation methods over traditional approaches.

## 5 Conclusion

The use of ML models combined with advanced validation techniques, such as bootstrapping and extended calibration evaluation represents a novel approach in the context of readmission prediction. These underutilized methods offer enhanced model performance and reliability.

The lack of studies of this type in our region makes this research a pioneering effort in Latin America. Future research should focus on external validation and the integration of these models into clinical workflows to maximize their impact on patient care and healthcare system performance.

## Supplementary Material

vbaf121_Supplementary_Data

## Data Availability

Data available on request.
