# Supplementary figures and images for: Predictive machine learning model for 30-day hospital readmissions in a tertiary healthcare setting
Source: Bioinform Adv. 2025 May 24;5(1):vbaf121. doi: 10.1093/bioadv/vbaf121 (PMC12158157; doi:10.1093/bioadv/vbaf121)

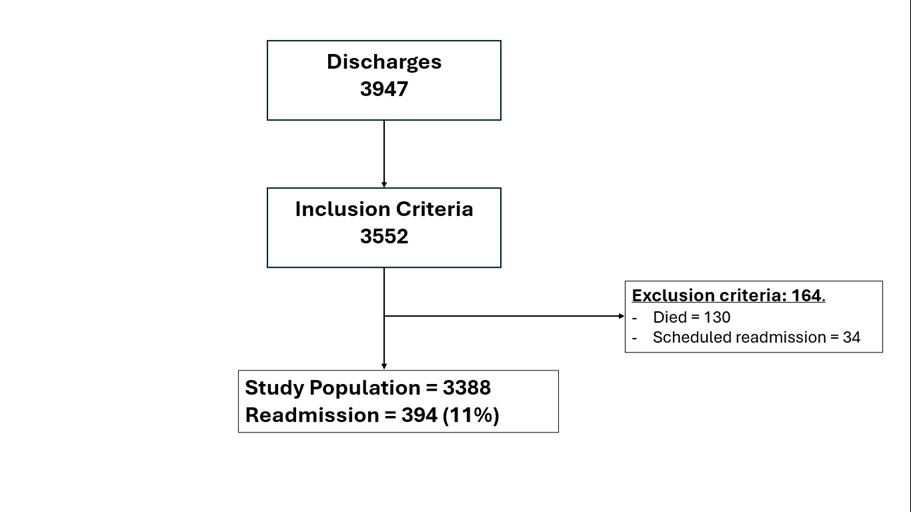

Supplement: vbaf121_Supplementary_Data [file vbaf121_supplementary_data.zip › Supplement Figure 1.jpg]

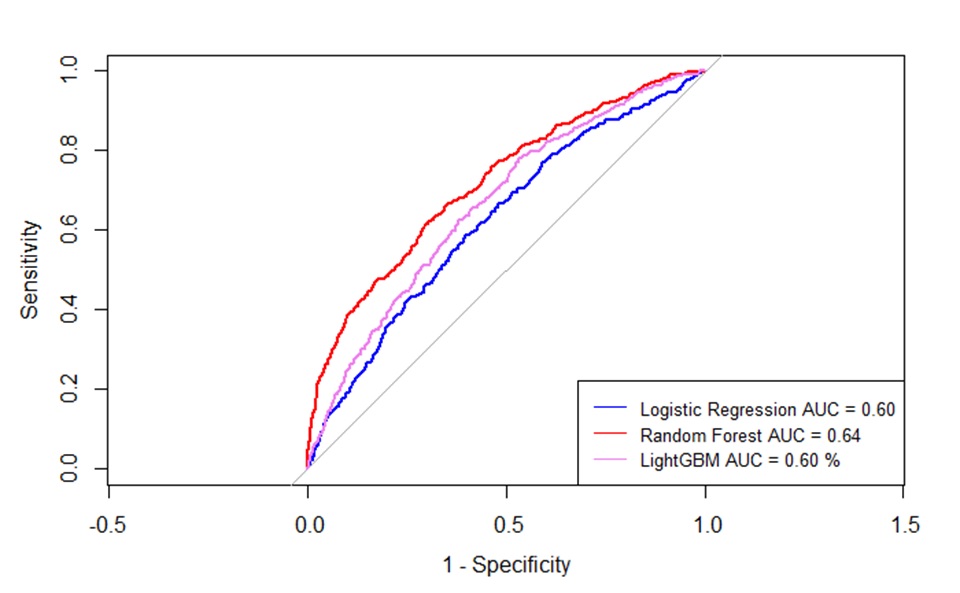

Supplement: vbaf121_Supplementary_Data [file vbaf121_supplementary_data.zip › Supplement Figure 2.jpg]

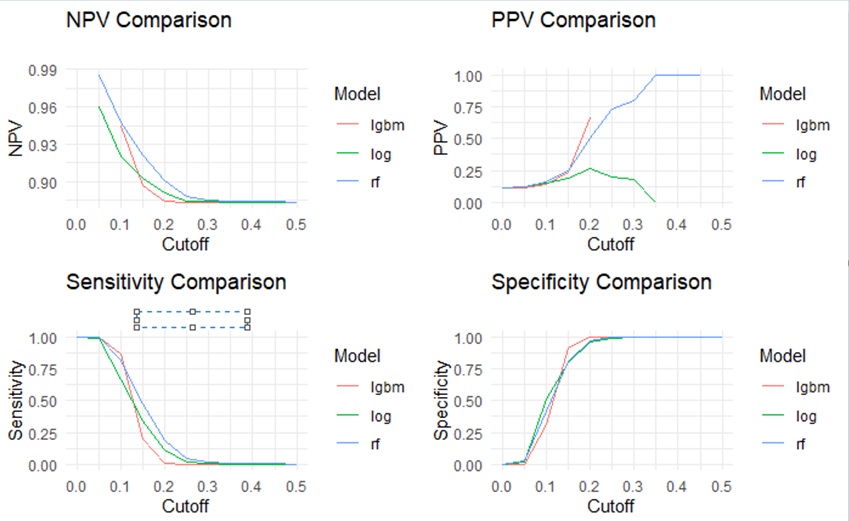

Supplement: vbaf121_Supplementary_Data [file vbaf121_supplementary_data.zip › Supplement Figure 3.jpg]

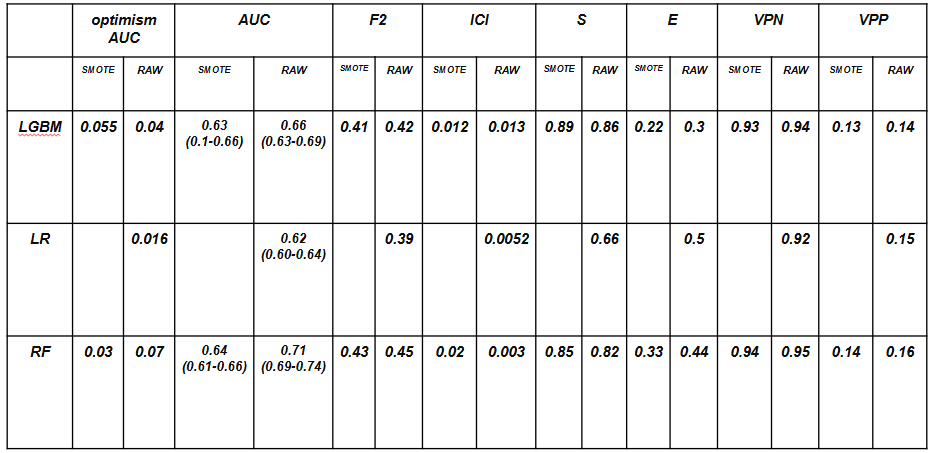

Supplement: vbaf121_Supplementary_Data [file vbaf121_supplementary_data.zip › Supplement Table 1.png]
